# *Candidozyma auris* Outbreak and Its Effective Control in a General Hospital

**DOI:** 10.3390/antibiotics14060579

**Published:** 2025-06-05

**Authors:** Flora V. Kontopidou, Maria Antonopoulou, Anastasia Votsi, Vassiliki Papoutsaki, Vassiliki Bereri, Evangelia Kourkoulou, Amalia Rompola, Georgia Tsokou, Anna Pavli, Olga Maria Koutantelia, Maria Siopi, Sevasti Leventaki, Joseph Meletiadis, George L. Daikos

**Affiliations:** 1 Infection Control Committee, Mitera General Hospital, 15123 Athens, Greece; fkontopid@yahoo.gr (F.V.K.); mantonopoulou@mitera.gr (M.A.); olga_volos08@yahoo.gr (O.M.K.); 2 Clinical Microbiology Laboratory, Mitera General Hospital, 15123 Athens, Greece; anastasiavo49@gmail.com (A.V.); vassiliki.papoutsaki@gmail.com (V.P.); 3 Nurse Department, Mitera General Hospital, 15123 Athens, Greece; vassobereri@gmail.com (V.B.); evangeli_kourkoulou@hotmail.com (E.K.); apavli@mitera.gr (A.P.); 4 Intensive Care Unit, Mitera General Hospital, 15123 Athens, Greece; arompola@mitera.gr (A.R.); georgia.tsokou@yahoo.gr (G.T.); 5 Clinical Microbiology Laboratory, National and Kapodistrian University of Athens, Attikon University Hospital, 12462 Athens, Greece; marizasiopi@hotmail.com (M.S.); sevyleve@gmail.com (S.L.); jmeletiadis@med.uoa.gr (J.M.)

**Keywords:** *C. auris*, healthcare-associated infections, active surveillance, infection prevention and control

## Abstract

**Background/Objectives:** *Candidozyma auris* (*C. auris*) is a multidrug-resistant pathogen recognized as a serious global public health threat. Herein, we report a *C. auris* outbreak that was successfully contained in a general hospital located in Athens, Greece. **Methods:** This study was conducted between December 2021 and December 2023. Upon identification of the first imported case of *C. auris*, the following infection control measures were applied in a stepwise approach: the promotion of hand hygiene, contact precautions and isolation, chlorhexidine gluconate bathing of patients, enhanced environmental cleaning, and active surveillance cultures of all high-risk patients upon admission. Active surveillance cultures were performed weekly in non-colonized ICU patients. **Results:** A total of 1564 screening samples from 890 patients were analyzed. Sixty-three patients were identified to be colonized and three to be infected with *C. auris.* After implementing screening and enhanced environmental cleaning, the quarterly incidence of hospital-acquired cases decreased from 0.37 to 0.04 cases per 1000 patient-days (slope of linear trend: −0.08; 95% CI: −0.16 to −0.0004; *p* = 0.05), despite the continuous inflow of already colonized patients. **Conclusions:** A bundle of infection control measures, including active surveillance cultures upon admission and enhanced environmental cleaning, can contain *C. auris* dissemination in acute healthcare settings.

## 1. Introduction

*Candidozyma auris* (*C. auris*) is a drug-resistant pathogen recognized as a serious global public health threat by both the CDC and the WHO [1,2,3]. After the initial report in Japan in 2009 [4], *C. auris* has spread across many countries in all six major continents. Genetic analysis of the species has revealed six clonal lineages (I to VI) with distinct geographic clustering: the South Asian clade I, the East Asian clade II, the African clade III, the South American clade IV, and the Iranian clade V. A sixth clade has recently been described in Singapore and Bangladesh in the Indomalayan zone, showing a close relationship with clade IV [5]. Global warming may have enabled *C. auris* to adapt to higher temperatures, allowing it to cross the “endothermy barrier” and become a human pathogen [6]. This organism has the ability to colonize human skin, adhere and persist to inanimate surfaces (a process that is facilitated by the fungus adhesin surface colonization factor (Scf1)), and cause difficult-to-control outbreaks [7,8,9]. More importantly, after skin colonization, *C. auris* may result in invasive infections, primarily candidemia and/or deep-site infections via hematogenous spread to distal anatomical sites [4]. Furthermore, the increased resistance rate of *C. auris* to major antifungal agents, combined with difficulties in laboratory detection and the frequent misidentification of *C. auris* as other *Candida* species [10,11], pose substantial challenges to treatment efficacy and to the prompt implementation of appropriate infection control measures [12,13,14,15].

In Greece, the first confirmed case of *C. auris* was reported by public health authorities in 2019 [16]. Since then, numerous cases have emerged in healthcare facilities across the country, with a marked increase during the COVID-19 pandemic [17,18,19,20]. The wide spread of *C. auris* in the Attica region, Greece’s largest healthcare district, has been linked to several factors, such as repeated hospital readmissions of chronic patients, extended hospital stays, and delays in identifying carriers, all of which have contributed to insufficient infection control practices and the further spread of the pathogen within hospital settings [21]. These challenges, coupled with Greece’s endemic situation involving carbapenem-resistant Gram-negative pathogens [22], have created an exceptionally difficult scenario to manage. The situation is further complicated by shortages of single rooms for isolation and healthcare personnel.

This report outlines the intervention strategies that successfully contained the spread of *C. auris* in a general hospital and addresses the challenges faced in managing this public health crisis.

## 2. Results

**Figure 1:**
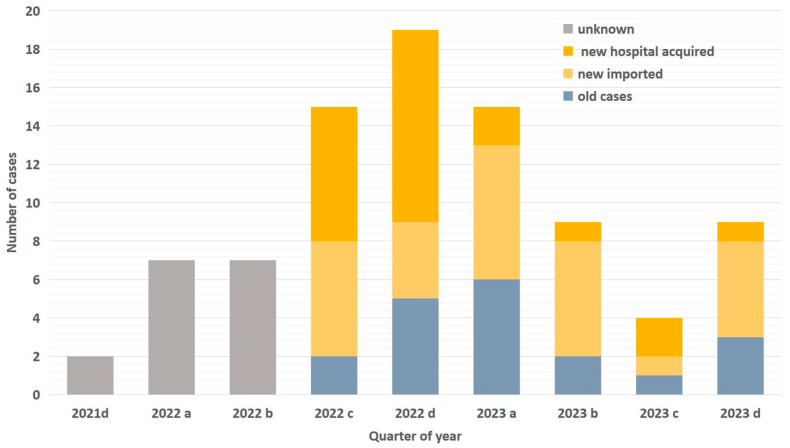
Number of patients with *C. auris* per quarter. Each quarter depicts all hospitalized patients with *C. auris* (new cases (imported or hospital-acquired), unknown cases, and old cases consisting of those who remained in the hospital for a period longer than the quarter in which they were first detected). The letters a, b, c, and d indicate the respective quarter of the year.

Patients: A total of 1564 samples were collected from 890 patients. Sixty-three patients were identified to be colonized and three to be infected with *C. auris*. As shown in Table 1, the majority of patients (73%; n = 48) were ICU patients, while the remaining 27% (n = 18) were hospitalized in medical or surgical wards. In total, 14 patients were detected prior to the initiation of active screening, while 52 were identified after the initiation of active screening, 29 upon admission (3.4% of patients screened), and 23 during their hospital stay (Figure 1). The first positive culture for *C. auris* was derived from axillae–groin swabs in 46 patients (70%), from rectal swabs in 10 (15%), from urine samples in 5 (7.5%), from blood in 3 (4.5%), and from bronchial secretions in 2 (3%). The 11 patients identified from clinical samples were all found to have skin colonization upon subsequent screening. The median time from hospital admission to colonization or infection was 16 days (range: 11 to 66 days). Among the 66 colonized/infected patients, 38 were males and 28 were females, with a median age of 73 years. All patients had at least one comorbidity (Table 1). The median Charlson’s comorbidity index was 5 (IQR: 4, 6). The median length of hospitalization was 30.5 days (range: 4–230 days), and the all-cause in-hospital mortality rate was 27% (18 out of 66 patients died).

In total, 3 of 66 patients (4.5%) developed candidemia, all of whom were ICU patients on mechanical ventilation with ages of 72, 75, and 78 years and APACHE II scores on admission of 7, 8, and 15, respectively. All had previously received antimicrobial and antifungal therapy with caspofungin. Candidemia was cleared within six days, and all had a good clinical response. One patient died before hospital discharge for reasons unrelated to candidemia.

Compliance with Intervention Measures: The average monthly compliance with hand hygiene among healthcare workers increased from 52% before the intervention period to 78% in the post-intervention period. A high level of adherence was observed in the implementation of isolation/cohorting (100% of colonized/infected patients) and in the application of contact precautions (100%). After the initiation of active screening upon admission, 100% of high-risk patients for *C. auris* were screened. Enhanced cleaning of the patient care environment was performed three times daily, and terminal disinfection with hydrogen peroxide was advised after patients’ discharge.

**Figure 2:**
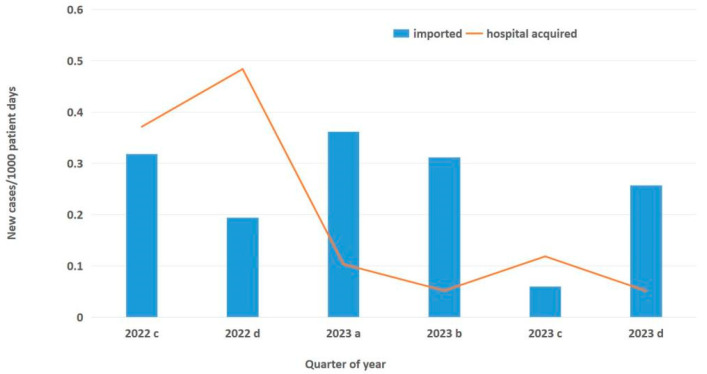
Quarterly incidence rate of new *C. auris* cases during post-intervention period. Columns indicate imported cases, whereas line indicates hospital-acquired cases. The letters a, b, c, and d indicate the respective quarter of the year.

Epidemic Curve and Incidence: As shown in Figure 1, the first case of *C. auris* was identified in December 2021. The number of cases progressively increased, but after the introduction of active screening and enhanced environmental cleaning, the number of cases started to decline. Despite a continuous influx of already-colonized patients during the post-intervention period, the quarterly incidence of hospital-acquired cases decreased from 0.37 to 0.04 cases per 1000 patient-days (slope of linear trend: −0.08; 95% CI: −0.16 to −0.0004; *p* = 0.05), indicating a significant reduction in ongoing transmission (Figure 2).

*C. auris* isolates: In vitro susceptibility data are shown in Table 2. All isolates were interpreted as non-resistant to amphotericin B, and echinocandins, while 36/49 (74%) were fluconazole-resistant. Despite the fact that most isolates exhibited a wild-type phenotype to other azoles, they may harbor resistance mechanisms, as most of them were fluconazole-resistant (Table 2). All isolates were confirmed to belong to South Asian clade I.

## 3. Discussion

The findings presented herein demonstrate that early detection of asymptomatic *C. auris* carriers, followed by separation from non-carriers, in conjunction with contact precautions and enhanced environmental cleaning, had a significant impact on containing the dissemination of this organism in our healthcare facility despite the constant inflow of colonized patients.

From the outset of the intervention, two main patient groups were identified as contributing to the introduction and spread of *C. auris* within our facility. The first group consisted of patients with prior hospitalization in acute or long-term healthcare facilities, and the second group included ICU patients with multiple comorbidities. These two patient groups became the target population of the infection control program implemented in our hospital. High-risk patients were prioritized for screening upon admission, and the colonized or infected patients were subjected to rigorous infection control practices throughout their hospital stay.

Implementing this comprehensive bundle of infection control measures across different clinical areas to prevent the spread of the pathogen within the hospital presented significant challenges [25,26,27]. Ensuring full compliance with these infection control protocols, together with the ongoing support and monitoring of the program in the wards and maintaining open communication between the patient-receiving departments to keep all staff informed of the necessary measures, was critical to the success of the intervention. Each patient with *C. auris* served as a powerful reminder to the entire hospital of the need for strict adherence to prevention and control measures. This communication extended to the Infection Control Committee and senior clinical and administrative leadership within the hospital. The awareness and commitment of the clinical leadership and hospital administration were critical to the effective implementation of these measures, as highlighted by international best practices in managing similar crises within healthcare settings [28,29].

Prior to the intervention, a substantial proportion (40%) of ICU patients were colonized with *C. auris*. This appeared to be the result of (i) the inflow of colonized patients not recognized upon admission and (ii) ongoing transmission from patient to patient or from the inanimate environment to patients via the hands of healthcare workers due to inadequate infection control practices. In healthcare facilities with such characteristics, the early detection of carriers and separation from other patients are crucial to contain the spread. During the outbreak, efforts were made to relocate all affected patients to a designated ward with medical equipment dedicated to individual patients. We attempted to implement the same strategy in the ICU, where patients were hospitalized in single rooms or isolation boxes. However, cohorting patients and staff in the ICU posed significant challenges due to patient colonization with other multidrug-resistant organisms. Daily planning was necessary to group patients and assign nursing staff based on colonization status.

Clinical cultures failed to detect a significant proportion of colonized patients, and unrecognized carriers can serve as a potential source of transmission to other patients. Active surveillance for the identification of asymptomatic carriers has been recognized as an effective strategy to contain the spread of contact-transmissible pathogens in hospitals, especially in high-risk areas such as ICUs [30,31,32]. In accordance with these observations, the incidence of colonization in the present study began to decline only after employing active surveillance cultures. Our findings emphasize the need for prompt implementation of such measures to reduce the risk of exposing other patients. Isolation and contact precautions should be implemented in all high-risk patients immediately on admission to the hospital, and decisions about whether or not to continue them can be made when the results of surveillance cultures are available. However, even after employing active screening and the intensification of contact precautions and the isolation of all colonized/infected patients, and despite improvements in hand hygiene compliance, *C. auris* has not been completely eliminated, and cases continue to occur. This probably reflects the persistence of this fungal pathogen in the inanimate hospital environment.

This hypothesis was supported by an ICU patient who had tested negative upon admission but was found to be colonized during his hospital stay, despite the fact that there were no colonized or infected patients with *C. auris* in the unit at that time. It is possible that this particular patient acquired *C. auris* from the environment or from reusable medical equipment that remained contaminated from the previous patient. This event prompted the infection control committee to intensify environmental cleaning. It is worth noting that we only began to effectively control the outbreak after we had implemented enhanced environmental cleaning, particularly of reusable medical devices and “high-touch” areas. These findings are in line with previous studies that emphasize the critical role of environmental contamination in the spread of *C. auris* in healthcare settings [33,34,35]. Notably, in a recent outbreak in a neuroscience ICU, the survival of *C. auris* in reusable equipment, and, in particular, in axillary thermometer probes, appeared to explain the persistent transmission of this fungal pathogen [36]. A major concern is the ability of the pathogen to form layers of biofilms that are resistant to standard hospital disinfectants, allowing it to persist on surfaces for extended periods of time.

*C. auris* is a notorious fungal pathogen exhibiting an often multidrug-resistant phenotype. Antifungal susceptibility testing revealed that 74% of our isolates were resistant to fluconazole, but none of the isolates were resistant to echinocandins or amphotericin B. All 49 isolates that were examined in the present study belonged to the South Asian clade I, as did the isolates from other hospitals in Greece [19,21]. Apparently, the dissemination of *C. auris* in our geographical region arose from the introduction of South Asian clade I, which subsequently spread across many hospitals. It is worth noting that invasive infections occurred in only three patients, all of whom were successfully treated with echinocandins, and no deaths were directly attributed to *C. auris*.

Our findings should not be interpreted without considering several limitations. First, the design of the present study did not allow us to assess the effectiveness of each intervention in controlling the outbreak. However, the findings underline the importance of active screening and environmental cleaning in conjunction with the other infection control measures. Second, this was a descriptive study, and no robust statistical analysis was performed to identify risk factors associated with *C. auris* colonization or infection.

In conclusion, our results indicate that a multifaceted intervention program, including active surveillance and enhanced environmental cleaning, with a focus on reusable medical devices and “high-touch” areas, can achieve significant control of *C. auris* outbreaks in acute healthcare settings. The value of active surveillance is even greater in healthcare units that regularly admit patients colonized with this fungus from high-prevalence settings. Ensuring compliance with these measures is a complex process and requires continuous vigilance, monitoring, and adaptation of the protocol in the hospital setting.

## 4. Materials and Methods

Setting: This study was conducted between December 2021 and December 2023 in a 500-bed general private hospital located in Athens, Greece, serving the adult and pediatric populations. The hospital consisted of eleven wards and three mixed ICUs (one for neonates, one for children, and one for adults). The mean nurse-to-patient ratio in the wards was 1:10 during the morning shift and 1:15 during the afternoon and night shifts, and in the ICUs, it was 1:2 in the morning shift and 1:3 in the other two shifts. The first case of *C. auris* in our hospital was identified in December 2021 from a urine sample of an adult ICU patient who was transferred from another healthcare facility. The same month, a second case of *C. auris* was identified in the same unit, and a point prevalence survey of skin, urine, and rectal colonization was conducted among adult ICU patients, which revealed that four of ten patients were colonized with *C. auris*. The identification of more than one patient colonized with this pathogen, which had not previously been detected in our hospital, with epidemiological links between the patients in terms of time and place, was considered and managed as an outbreak. These observations prompted the infection control committee to design and implement an enhanced infection control program.

**Figure 3:**
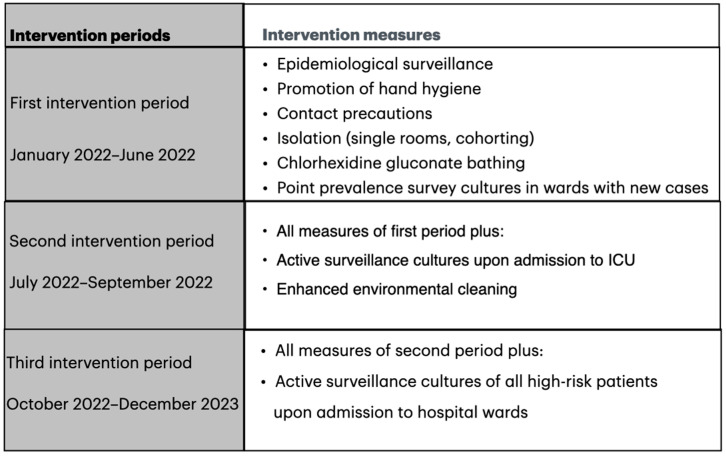
Intervention measures according to time periods.

Intervention: Before implementing the infection control measures, a training program was conducted in the hospital, aiming to improve healthcare workers’ compliance with hand hygiene protocols. Training and feedback were provided by the infection control team once a month initially and every two months thereafter. Alcohol-based disinfectants were located near all patient beds. Personal protective items, including gloves and gowns, as well as dedicated medical equipment (e.g., stethoscopes, thermometers, and blood pressure manometers), for every individual patient’s use, were made available in every room of colonized or infected patients. The infection control measures were applied in a stepwise approach, as shown in Figure 3. At the beginning of the intervention (January 2022–June 2022), the basic infection control measures were implemented according to national and global guidelines, including the promotion of hand hygiene, contact precautions, isolation in single rooms or cohorts, and the chlorhexidine gluconate bathing of colonized or infected patients [25,26,27]. Furthermore, point prevalence survey cultures of skin (axillae and groins) and rectal swabs were obtained from all patients in the same ward when a new case of *C. auris* was identified. In the second period of intervention (July 2022–September 2022), in addition to measures of the first period, active surveillance cultures of skin, bronchial secretions, urine, and rectal swabs for *C. auris* were obtained from all adult ICU patients upon admission and weekly thereafter for those who were negative in the first screening. Also, the frequency of environmental and medical equipment cleaning was increased from two to three times/day, with a special emphasis on the cleaning and disinfection of high-touch surfaces and reusable medical devices (e.g., monitoring devices, dialysis machines, electrocardiographs, and ultrasound machines). After the discharge of a colonized/infected patient, thorough mechanical cleaning and disinfection with hydrogen peroxide were performed in the patient’s room. In the third period (October 2022–December 2023), active surveillance cultures of skin and rectal swabs were extended to all high-risk patients (those with prior hospitalization for more than 48 h over the last year, and residents of long-term care facilities) upon admission into the hospital wards. All high-risk patients remained in isolation under contact precautions until the culture results were available. Periodically, environmental cultures were obtained to validate the effectiveness of the cleaning process.

Monitoring for Compliance: Hand hygiene compliance was monitored by unobtrusive observations of patient–healthcare worker contacts. A trained observer recorded the opportunities for hand hygiene and the action performed by the HCW as either action performed (rubbing with an alcohol-based hand rub, washing with soap and water, or both washing and rubbing) or not performed according to “My five moments for hand hygiene” of the WHO Hand Hygiene Improvement Strategy. A mean of 800 observations were performed every month. Compliance with the isolation of colonized/infected patients (in single rooms or cohorts), as well as the implementation of contact precautions, was also monitored daily.

Epidemiological Surveillance: Patients colonized or infected with *C. auris* were included in the hospital’s mapping of pathogens of critical concern. Pertinent information, including demographic characteristics, underlying diseases, Charlson’s comorbidity index [37], prior hospitalizations, prior exposure to antifungal agents, microbiological data, the treatment of candidemia, and outcomes, was extracted from patients’ medical records in a predesigned form. Each new case of *C. auris* was categorized as imported (positive cultures upon admission), hospital-acquired (negative cultures upon admission and a positive culture any time after admission), or of unknown origin (no screening upon admission and positive culture during hospitalization).

Microbiology: The screening for *C. auris* colonization was based on culture methods. Swabs were obtained from patients’ axillae and groin areas and rectum, as well as other sites, if clinically indicated. The specimens were transferred to the laboratory in transport media. Following enrichment in thioglycolate broth and incubation at 37 °C for 24 h, the samples were inoculated on CHROMID^®^ chromogenic agar plates (bioMerieux, Marcy-l’Etoile, France) and incubated at 37 °C for a minimum period of 2 days. Pale pink yeast colonies grown on agar plates were identified by matrix-assisted laser desorption/ionization time-of-flight mass spectrometry (VITEK MS, bioMerieux, Marcy l’Etoile, France). All isolates were stored in normal sterile saline with 10% glycerol (AppliChem, Darmstadt, Germany) at −70 °C for further examination. Prior to testing, they were sub-cultured on in-house prepared antimicrobial-free Sabouraud dextrose agar (Oxoid, Athens, Greece) plates at 35 ± 2 °C for 24 h.

Susceptibility Testing: The in vitro susceptibility to amphotericin B, anidulafungin, micafungin, caspofungin, fluconazole, voriconazole, posaconazole, itraconazole, and isavuconazole was assessed according to the Clinical and Laboratory Standards Institute [CLSI] protocol M27-Ed4 [38]. The susceptibility data were interpreted according to the Centers for Disease Control and Prevention’s (CDC) tentative resistance breakpoints for *C. auris* where available, i.e., fluconazole ≥ 32 mg/L, amphotericin B ≥ 2 mg/L, anidulafungin ≥ 4 mg/L, micafungin ≥ 4 mg/L, and caspofungin ≥ 2 mg/L, whereas for drugs without breakpoints, previously proposed species-specific CLSI epidemiological cutoff values were used [23,24]. Thus, isolates were classified as non-resistant or resistant to fluconazole, amphotericin B, and echinocandins, and the wild type or non-wild type to the other antifungals.

Clade Typing: A five-allele-specific polymerase chain reaction assay for the identification of *C. auris* and each of its five major described clades (I to V) based on conserved mutations in the internal transcribed spacer rDNA region and a clade-specific gene cluster was used as previously described [39]. Five strains, one from each different clade, were kindly provided by Jacques Meis (CWZ Hospital, Nijmegen, The Netherlands) and used as controls.

Statistics: We estimated the incidence rate of the hospital acquisition of *C. auris* colonization/infection per quarter of the year. The incidence rate was estimated as the number of new colonization/infection occurrences during hospitalization divided by the total patient-time at risk for that one-quarter of the year period. We also assessed the post-intervention slope of the linear trend in the quarterly incidence rate using linear regression.

## Figures and Tables

**Table 1:** Characteristics of patients colonized or infected with *Candidozyma auris*.

Characteristic	Number (%)
Age, median (IQR), years	73 (64, 78)
Sex Male Female	38.0 (57.5) 28.0 (42.4)
Ward ICU Non-ICU	48.0 (72.7) 18.0 (27.3)
Hospital-acquired Non-hospital acquired (identified upon admission) Unknown	23.0 (34.8) 29.0 (44.0) 14.0 (21.2)
Time from admission to colonization, median (range), days	16.0 [11–66]
Length of hospitalization, median (range), days	30.5 [4–230]
Central vascular catheter and/or urinary catheter	45.0 (68.1)
Mechanical ventilation	22.0 (33.3)
Prior use of antimicrobial agents	57.0 (86.3%)
Prior use of antifungal agents	22.0 (33.3)
Charlson’s comorbidity index, median (IQR)	5.0 (4, 6)
Myocardial infarction	7.0 (10.6)
Congestive heart failure	21.0 (31.8)
Peripheral vascular disease	21.0 (31.8)
Cerebrovascular accident	10.0 (15.5%)
Dementia	5.0 (7.5%)
Chronic pulmonary disease	14.0 (21.2%)
Peptic ulcer	1.0 (1.5%)
Liver disease	1.0 (1.5)
Diabetes mellitus	5.0 (7.5%)
Hemiplegia	4.0 (6.0%)
Chronic kidney disease	17.0 (25.7%)
Renal replacement therapy	12.0 (18.2)
Solid tumor	15.0 (22.7%)
Hematologic malignancy	2.0 (3.0)

**Table 2:** In vitro susceptibility profiles of 49 *C. auris* isolates to nine antifungals.

Antifungal Agent	Modal (Range) MIC (mg/L)	MIC_50_ (mg/L)	MIC_90_ (mg/L)	Non-R ^a^		R ^a^		WT ^b^		Non-WT ^b^	
				No (%)		No (%)		No (%)		No (%)	
Anidulafungin Caspofungin Micafungin Isavuconazole Fluconazole Itraconazole Posaconazole Voriconazole Amphotericin B	0.03 (0.016–1) 0.5 (≤0.06–1) 0.03 (0.016–2) ≤0.008 (≤0.008–0.125) 32 (4->64) 0.016 (≤0.008–0.5) ≤0.008 (≤0.008–0.125) 0.125 (0.016–2) 1 (0.5–1)	0.03 0.5 0.03 ≤0.008 32 0.03 ≤0.008 0.125 1	0.125 0.5 0.125 0.03 >64 0.125 0.06 0.5 1	49 49 49 - 13 - - - 49	(100) (100) (100) - (26) - - - (100)	0 0 0 - 36 - - - 0	(0) (0) (0) - (74) - - - (0)	- - - 49 - 47 49 48 -	- - - (100) - (96) (100) (98) -	- - - 0 - 2 0 1 -	- - - (0) - (4) (0) (2) -

**^a^** Based on CDC: Centers for Disease Control and Prevention [23]; **^b^** Based on ECVs: epidemiological cutoff values [24]; MICs: minimum inhibitory concentrations; R: resistant; WT: wild type.

## Data Availability

Data are contained within the article.
